# Changing Directions and Expanding Horizons: Moving towards More Inclusive Healthcare for Parents of Children with Developmental Disabilities

**DOI:** 10.3390/ijerph20216983

**Published:** 2023-10-27

**Authors:** Monika Novak-Pavlic, Peter Rosenbaum, Briano Di Rezze

**Affiliations:** 1 School of Rehabilitation Science, McMaster University, Hamilton, ON L8S 1C7, Canada; direzzbm@mcmaster.ca; 2 CanChild Centre for Childhood Disability Research, McMaster University, Hamilton, ON L8S 1C7, Canada; rosenbau@mcmaster.ca

**Keywords:** childhood disability, family well-being, family support, parenting, parents of children with disabilities, pediatric rehabilitation

## Abstract

Family-centred service (FCS) acknowledges the importance of family engagement in therapeutic processes and focuses on the needs of all family members. This way of thinking and practicing is becoming increasingly recognized as an optimal care delivery model for families of children with developmental disabilities (DDs). However, in most places, disability services are oftentimes ‘child-centric’, wherein family members are seen only as partners in therapy or care delivery, while their own needs are not addressed. This arises from the lack of awareness of complex and highly individual family needs by professionals with whom they interact, but also from a significant lack of service infrastructure oriented towards parent-specific needs in existing service delivery models. This concept paper highlights the known challenges associated with parenting a child with a DD and discusses the intersectionality of factors impacting parental health and well-being, with a goal of promoting more equitable, holistic, and inclusive healthcare for all family members of children with DDs.

## 1. Childhood Developmental Disability: Then and Now

Since the end of the 20th century, we have been witnessing a strong movement to deinstitutionalize children living with a life-long developmental disability (DD) [1,2]. Prior to this change, the somewhat naïve belief was that children whose development was not considered ‘typical’ should be moved away from their nuclear family and live in an institution where professionals of various disciplines were supposed to ‘fix’ their disability and prepare them for independent living [1]. An underlying assumption was that the real-life experiences these children would otherwise have in their families lacked the quality or quantity needed to support their development. The way we view childhood disability has changed dramatically in the last two decades [3,4,5,6]. Depending on local political and societal efforts to discontinue the institutionalization of children with disabilities, many developed countries have now fully or almost fully transitioned to family-based alternatives, with children with impairments living with their nuclear or extended family [2,7].

As a result of these changes, the responsibility for a child’s development and for navigating the services they need has now fully fallen on their caregivers. This change has also challenged the way clinicians and researchers think and talk about childhood DDs. The predominant paradigm until the end of the 20th century—the *medical model* rooted in a biomedical approach to disability—guided both parents’ and professionals’ actions. This thinking conceptualized disability or disease as an absence of health that should be prevented, treated, fixed, or at least minimized [8,9]. This reductionist polarized model, based on fundamentally faulty assumptions, has proved lacking. The idea that a child’s functioning can be fully seen and explained as either ‘normal’ or ‘abnormal’ seemed (and in our view was and is) oversimplified [10]; rather, there has been a gradual recognition that other factors have important contributing impacts on children’s health and developmental outcomes. There was a clear need for a new conceptual model that would support the importance of personal, psychological, and environmental influences on an individual’s health.

**Figure 1:**
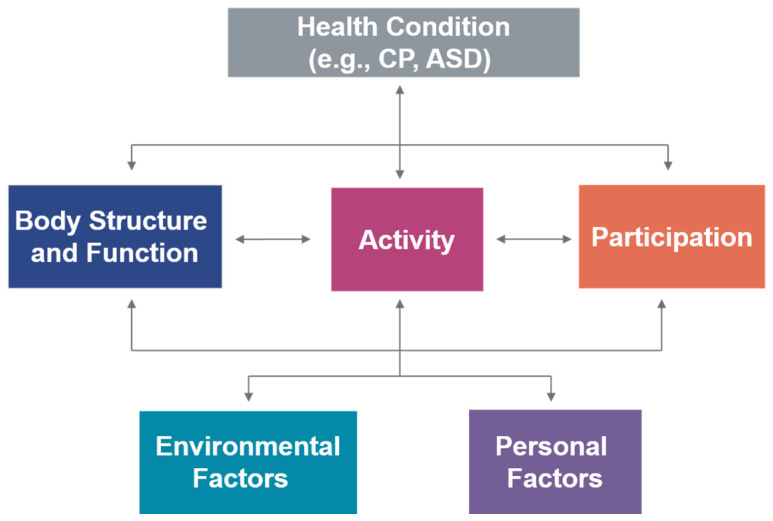
International Classification of Functioning, Disability and Health (WHO, 2001). From CanChild (https://www.canchild.ca/en/research-in-practice/f-words-in-childhood-disability/icf-resources (accessed on 11 September 2022). Copyright 2021 by McMaster University.

In 2001, the World Health Organization (WHO) released the International Classification of Functioning, Disability and Health (ICF), a revised and expanded version of the 1980 International Classification of Impairments, Disabilities and Handicaps [10]. This framework for health aimed to provide a comprehensive and holistic biopsychosocial conceptual model for describing health and disability. According to the ICF, a person’s health status or condition is a product of the interaction between their body functions and structures, activities, and participation, all of which are under the strong influence of personal and environmental factors (Figure 1) [10]. This universal framework is based on each person’s strengths and not on their ‘lack of abilities’. The ICF framework acknowledges that there are many contributing and interconnected factors impacting someone’s health, including environmental and personal factors [10].

With this expanded view on how we describe health and disability, parents of children with DDs and professionals in pediatric healthcare have been given a powerful framework to guide discussions, identify the family’s and child’s strengths, facilitate setting goals, and help discover the areas in which they require support [11]. Note that by seeing the *family* as the unit of our focus, we encourage and enable parents to include the needs of all members, including other children, in the discussion. Siblings’ lives as developing people, and as people in the role of ‘sibling’, are recognized to be as important as the needs of the index child with the complex medical or developmental needs that bring the family into our orbit. The ICF paradigm, built on the biopsychosocial perspective on health, has been widely welcomed as a new and expanded inclusionary way of thinking that is currently considered the most comprehensive and appropriate model for discussing health and disability, including childhood DDs [12].

With children growing up in their own families, caregivers of children with DDs—in most cases their parents—are expected to take on caregiving demands while continuing to manage their other duties (e.g., household chores, work). Parents of children with DDs today are expected not only to provide a loving and caring environment for their children but also to navigate complex healthcare and education systems while ensuring balanced and supportive development of their child. While deinstitutionalization has brought many positive changes to how children grow and develop, it sometimes comes at a cost to parental health, well-being, and prosperity.

We know that the lives of parents and families of children with DDs are usually more complicated than the lives of parents who raise typically developing children. In many high-income countries, parents of children with DDs can receive various types of medical, financial, or instrumental support to help them with the challenges they face. However, such support is often limited and insufficient and is usually directed specifically at interventions for the child and not the family [13]. There is a growing consensus that there are significant gaps in how we support parents in their parenting role [13,14]. Many of the challenges that parents face are based, at least in part, on how healthcare professionals and individuals without the same lived experience *think* and *talk* about childhood disability. The goals prioritized by *professionals* may not always align with those of the family or may not be easily integrated into a day-to-day family routine [13,15,16]. While therapists traditionally focus on ‘body-oriented’ activities, parents and children might be more interested in participation skills that are transferable to their daily environment [15]. This disconnect can create an additional burden on the already stressful realities of parents whose child happens to have a life-long DD impacting their everyday functioning. Additionally, parents encounter stigmatization in their immediate and extended social circles due to their child’s disability. This ‘undesired differentness’ [17] (p.43) manifests via discrimination, labelling, stereotypes, and social isolation directed either towards the child or the family unit [17,18].

Based on the literature, a better alternative to the deficit-focused approach is the strength-based, holistic way of thinking that can be protective of families whose experience of being a parent to a child with a DD might have been framed negatively [19,20,21]. Building on the available literature that points out the gaps in parent support, as well as the clinical gap in addressing parents’ needs, we are urged to find ways to develop and promote positive and holistic practices to support parents of children with DDs in the context of pediatric developmental rehabilitation services built on the ICF ideas.

## 2. Raising a Child with a Developmental Disability: State of the Evidence

Being a parent is a demanding and ever-changing role. In situations where a child’s health or development is disrupted or not considered ‘typical’ for age, the trajectory of parenting can take a major shift that impacts the entire family. Parents can be under extreme stress and experience increased demands to ensure that their child is receiving the medical attention they need [22,23,24]. When a family receives a diagnosis that their child has a life-long illness or disability, this often means a significant change in life circumstances for the family, along with the need for emotional coping [22,23,25,26]. Medical procedures, tests, therapies, and frequent or prolonged hospital stays may all be imposed on parents and represent the reality of raising a child with a disability. Difficult life circumstances and overwhelming demands that are placed on them are sometimes beyond what parents have expected or wished for themselves or their children. Parents can feel like they have lost control over their lives as they learn how to navigate the world of childhood disability [27].

Being a parent to a child with a disability can also be a fulfilling experience. In recent years, there has been growing interest in exploring the transformative, positive elements of parenting a child(ren) with developmental challenges that many parents of children with disabilities experience [28,29,30,31]. A study by Skotko et al. (2011) revealed that 79% of parents of children with Down syndrome (DS) (*n* = 2044) believed that their view on life had become more positive because of their child with DS [32]. Similar results were found in a small sample of parents of children with various disabilities [28]. The majority of parents in the study talked about the positive characteristics of their child and cherished their child’s achievements. Most parents believed that their child’s disability positively changed their perspective on life and increased their sensitivity, tolerance, patience, and awareness of others [28]. A qualitative study by Beighton and Wills (2017) showed that all participants in their study felt that they “had become stronger, tougher and more confident” [25] (p. 332). Parents also talked about gaining personal strength due to intense advocating and ‘fighting’ for their children. They also mentioned having a greater appreciation of life and celebrating their child’s accomplishments [25]. The positive effects of parenting were identified even when the predicaments of parenting were present [25,33], meaning that the positive and difficult sides of parenting a child with a disability can co-exist and are not mutually exclusive.

Despite the positive aspects of raising a child with a disability, we know that parents also face considerable challenges with their own health and well-being, some of which have been extensively explored in the literature. A systematic review by Cohn et al. (2020) assessed the health outcomes of parents of children with chronic illnesses across 26 studies [34]. The study showed that parents of children with chronic illness experienced poorer mental health when compared to parents of healthy children. Parents of children with chronic diseases were almost twice as likely to meet cut-off points for clinical depression and anxiety [34]. Similar results have been observed in other studies that specifically explored the health and well-being of parents of children with neurodevelopmental disabilities [35,36,37,38,39,40].

There is also evidence to suggest that parents’ physical health might be impacted when caring for a child with a developmental disability. A large Danish population-based cohort study by Cohen et al. (2018) assessed whether mothers of infants with a congenital anomaly (*n* = 42,943) had increased cardiovascular risks compared to mothers of infants with no anomaly (*n* = 428,401) [41]. Their research showed that mothers of infants with a congenital anomaly were more likely to suffer from cardiovascular disease (CVD), such as acute myocardial infarction, coronary revascularization, or stroke (adjusted HR (aHR) 1.15, 95% CI 1.07–1.23). CVD risk was even higher for mothers of infants with multi-organ anomalies (aHR 1.37, 95% CI 1.08–1.72) [41]. Another report conducted on the same population suggested that mothers of children with a congenital anomaly also had an increased risk of premature death due to cardiovascular disease, respiratory disease or other causes when compared to other mothers (aHR 1.22, 95% CI 1.15–1.29) [42]. A population-based prospective cohort study showed higher incidence ratios for the premature death of fathers of infants with a major congenital anomaly when compared with fathers of healthy infants born in the same time period (aHR 1.76, 95% CI: 1.64–1.88 vs. 1.62, 95% CI: 1.59–1.66). Apart from the previously mentioned physical health problems, there is also evidence that indicates that parents experience greater physical pain and activity limitations than their peers [38,39,43,44]. Other reported physical problems include a greater number of chronic conditions [37,38,39], migraines [38], stomach/intestinal ulcers [38], and poorer self-rated general health [35,37,39].

There are consistent data on the chronicity of increased stress levels for parents of children with disabilities [38,45]. A longitudinal study on maternal stress in childhood by Azad, Blacher, and Marcoulides (2013) showed that children’s characteristics, specifically behavioural problems and social skills, as well as mothers experiencing higher levels of stress in the child’s early childhood (3–5 years), were predictive of increased maternal stress in middle childhood (6–13 years) [45]. The positive correlation between the child’s behavioural problems and parents’ perceived stress has been widely reported in the literature [46,47,48].

Some studies indicated that mothers of children with disabilities might experience higher levels of stress than fathers [46,49,50,51]. Mothers’ higher levels of stress were associated with higher neuroticism scores, unemployment, unmet service needs and financial problems, while fathers’ stress was mostly associated with high neuroticism scores, not owning a car and poor family relationships [51]. Additionally, a study by Hastings et al. (2005) reported that mothers expressed significantly more depressive symptoms than fathers but also had a more positive perception of their child with autism [47]. These results suggest that there may be differences in perception of life and the impact of their child’s disability between mothers and fathers.

There are additional factors identified in the literature that appear to affect the health and well-being of parents. These include, but are not limited to, parents’ socioeconomic background [35,46], culture [29], religion [29], financial status [35,38], education level [35,46], social support [51,52,53,54], and quality of relationships [45].

A US population-based study by Kuo et al. (2011) explored caregiver challenges and showed that caregivers of medically complex children with special health care needs spent a median of 11–20 h per week providing direct home care for their child and two hours (IR 3–21+) per week coordinating their child’s care [55]. Many parents were forced to leave their jobs because of the care demands (54.1%) and experienced financial problems (56.8%) [55]. The financial and employment challenges, especially for mothers, have also been reported in other studies [38,46]. In addition, the estimated associated costs of having a child with autism were 8.4 to 9.5 times higher compared to parenting a child without autism [46], numbers that are similar to or even higher in other types of childhood disabilities. For example, according to the Centers for Disease Control and Prevention, medical costs are ten times higher for children with cerebral palsy (CP) than they are for children without CP [56].

A meta-analysis by Risdal and Singer (2004) looked at the prevalence of divorce among families of children with disabilities versus those without disabilities between 1975 and 2003 and found that parents of children with disabilities were more likely to divorce or separate (overall effect size d = 0.21) [57]. However, more recent studies detected no significant differences in divorce rates between parents of children with disabilities and typically developing children [58,59]. Evidence suggests that the likelihood of parental separation or divorce might differ depending on the type of disability. For example, a population-based study by Urbano and Hodapp (2007) showed lower divorce rates for parents of children with Down syndrome and other birth defects when compared to parents of children with no disability [60]. Based on the existing evidence, we can conclude that the chances that parents will experience marital problems might be higher on a population level or for some types of disabilities, but this is yet to be explored in robust, large-sample studies.

It is not surprising that such an intensive caregiving role, when accompanied by high costs, unemployment, and financial difficulties, might lead parents to experience a lack of control over their life circumstances. Although we are still unsure about the causal pathways of those events or how all these outcomes are interrelated, evidence shows that parents of children with disabilities have a higher chance of ‘secondary’ predicaments that are not directly related to their parenting or their child’s disability.

If parents are experiencing challenges with their physical health, psychological functioning or socioeconomic status, these predicaments will likely impact the entire family, including the child with a disability. Family adjustment to a child’s disability and overall functioning is also closely linked to the child’s developmental and health outcomes. However, parent–child interactions are reciprocal and transactional rather than simply unidirectional. When thinking about adequate and timely services for caregivers, it is reasonable to assume that such services can improve not just parent health outcomes but also the child’s. A review by Stein et al. (2014) showed that parental mental disorders were associated with their offspring’s mental disorders, emotional problems and externalizing behavioural difficulties [61]. Some studies from this review also demonstrated that children’s cognitive development, attachment to their caregiver, growth, and problems with weight (underweight, stunting, overweight, and body dissatisfaction) were negatively affected by the caregiver’s mental disorder. Given that we have only correlational data rather than causal connections between these outcomes, there are probably other contributing factors that also impact or cause such changes. The complexity and intersectionality of parents’ and children’s characteristics on family functioning have only indirectly been explored in the literature. A study by Garner et al. (2013) compared parenting behaviours across four groups of caregivers of children (those with (i) neurodevelopmental disorders, (ii) externalizing behavioural problems, (iii) neurodevelopmental disorder plus externalizing behavioural problems, and (iv) neither of these conditions) reported that caregivers of children with no health conditions had significantly higher positive interactions with their children and lower ineffective parenting behaviours compared to caregivers of children with disabilities or children with behavioural problems [62]. These results suggest that both children’s and parents’ characteristics reciprocally impact their relationship.

Most families of children with disabilities receive care or have contact with healthcare providers as part of pediatric healthcare services. Based on the evidence of the importance of family-centred service to families [63], those settings are ideal places to integrate strengths-based approaches, where parents can be informed not just about their child’s health or development but also about available resources and types of support that they might need immediately post-diagnosis or over time to prevent the potential risks to themselves and their family. Although still often overlooked in clinical practice, there is an increased recognition that parental well-being is a critical aspect of a family’s everyday functioning and prosperity. Many organizations around the world are developing and testing new programs for parents of children with disabilities that aim to promote connection, participation and belonging to local communities, all of which can act as protective factors in caring for a child with a DD. With this expansion of the research and practice focus in recent years, there has been a growing body of research on programs and initiatives that aim to address this knowledge-to-practice gap. This manuscript offers one such contribution by placing emphasis on parents’ and families’ needs via a research lens and highlighting the importance of expanding the focus from child-driven to family-centred approaches in pediatric developmental rehabilitation.

## 3. ‘It Takes a Village’, but Where Is It to Be Found

Parenting is oftentimes perceived as a natural stage in life that normally occurs within a specific time window for an individual. The phrase “It takes a village to raise a child” is believed to be an African proverb that refers to the idea that raising a child is not a task to be carried out by parent(s) alone [64]. The extended family, friends, neighbours, community, or so-called ‘villagers’ [64] are believed to all play an important role in the complete and healthy upbringing of a child. When thinking about parents of children with DDs, the parenting journey normally takes a different path than most parents anticipate because of the unexpected news about the child’s diagnosis and their additional needs (e.g., medical tests, specialist appointments, therapies). For many parents and families, this includes navigating extremely complex health, education, and social systems previously unfamiliar to them in order to secure a safe and supportive environment for their child.

It is well noted in the literature that parents of children with DDs can find navigating those worlds challenging or even overwhelming [65,66]. The need for a ‘village’ in circumstances like these is even higher than it would be for a parent or family of a typically developing child because of the increased and often unfamiliar care demands. Guided by this idea, one could assume that the ‘villages’ of children with DDs are larger, while in fact, many parents face social isolation and stigma in their local communities [67,68]. Indeed, one can offer the compelling argument that the modern ‘village’ should refer to our whole ‘society’, with social policies and services designed to support these parents and families as equally deserving members of the ‘village’ rather than as outsiders left to fend for themselves.

It would be incorrect to assume that parents’ struggles originate only from the fact that their child has a lifelong DD. Although most parents initially need time to process information about their child’s condition or diagnosis, many parents find ways to cope with the emotional heaviness and become more resilient [69,70,71]. On the other hand, the caregiving role is something that many parents continue to take on for the rest of their lives along with their own aging, which brings an issue of longevity (one might even say chronicity) of the caregiving role. Together with minimal external support to elevate some of the caregiver burden parents carry throughout their lives, this reality is an additional contributing factor to potential poor health and well-being outcomes over time. Despite the enormous body of evidence that identifies the specifics of parents’ experiences and potential adversities, parents are often not recognized as a vulnerable population. According to the National Collaboration Centre for Determinants of Health (n.d.), “vulnerable populations are groups and communities at a higher risk for poor health as a result of the barriers they experience to social, economic, political and environmental resources, as well as limitations due to illness or disability” [72]. The absence of ‘villages’ to alleviate the burden parents experience and societal changes in modern Western life have stimulated a series of shifts in structured systems, such as healthcare and social care. Many research and improvement initiatives aim to strengthen the amount and quality of services provided to individuals in need. In many ways, structured (government) systems have replaced ‘the village’, which has put pressure on structures that deliver care not only in terms of the quality of services but also the number of people who require them. Under those circumstances, coupled with the stress that has arisen from the COVID-19 pandemic, what can easily escape attention is an acknowledgement of the importance of *prevention* in the context of parental health rather than focusing on the treatment needed once a disease has already happened. When ‘villages’ are replaced by structured (often ‘sterile’ and soulless) environments, there are unavoidable shortcomings that arise from limited resources.

The issue of overlooked health and well-being of parents of children with DDs will likely not be solved via resource mobilization in one sector alone. Addressing the needs of families and helping them thrive requires not only transdisciplinary collaboration of professions but also an integration of fragmented systems and empowerment of communities. Safe and effective structured services can be an efficient way of targeting specific parental needs at the most sensitive time for receiving them. In addition, when creating strategies and planning parent empowerment initiatives, we should not omit the missing ‘village’. The demands greatly exceed currently available support services [73,74,75]. Particularly important to consider are the social determinants of health—non-medical factors such as income, employment, social inclusion, access to health care and others [10]—that are likely to have long-term health impacts on parents of children with DDs. The extent to which chronic social marginalization and disparities influence parent health and well-being outcomes remains unclear because of a lack of longitudinal tracking of parent-specific outcomes. So far, there has been little discussion about how we build meaningful preventative services and community-based resources grounded on family strengths. Instead of identifying risk factors and adversities when they have already happened, we could alternatively consider supporting parents at the start of their parenting journey to minimize the occurrence of undesirable outcomes that might appear at a later time. Such approaches - ‘early intervention for parents’ - might give parents tools to face difficult situations more successfully and find the support they need (for example, please see the ENVISAGE program [19,20,76]). In other words, shifting our perspective from risk to protective factors, and from child to child-and-family, can potentially be a cost-effective, preventative measure for supporting parental well-being.

## 4. Research and Practice Implications

With the growth of family-centred service and awareness of the need to support parents in their caregiving and care provision role in the applied health disciplines [14], there will continue to be high demand for timely, sensitive and (cost) effective interventions, specifically targeting parent-caregivers of children with DDs. Professionals working with children with DDs might find it challenging to connect with caregivers and be a source of support for their needs because of the lack of evidence and organization-specific protocols that would instruct their behaviour. The efforts to address this gap would, therefore, need to be multidimensional.

As more support programs continue to receive scholarly attention, the emphasis should be placed on the execution of high-quality, rigorous studies that can inform knowledge translation and practice recommendations. This, however, does not mean that only quantitative methodologies should be utilized. On the contrary, the breadth and depth of the complexity of parents’ personal experiences with multifaceted support programs are unlikely to be fully and fairly captured and represented with quantitative measures alone. When exploring the effects of (usually) complex interventions on latent constructs, ‘quantified’ data might have a limited ability to capture the intersectionality of all contributing factors. Qualitative data provide complementary rich descriptions of patients’ experiences that might sometimes represent the uniqueness of parents’ lives and perspectives more accurately. Quantitative and qualitative results complement each other and provide a more comprehensive and insightful view of underlying processes and effects, some of which often remain undetected in a solely quantitative study.

Currently, there is no gold standard for the provision of care for parents of children with DDs. We have a preliminary understanding of potential service delivery models and ways in which parents *could* be supported [14]. What remains unclear is what types of services are most acceptable and meaningful to families at various stages of their child’s life. As children are developing, families are also changing. Building the capacity of organizations and communities to keep up with personal (family-specific) and external (societal and environmental) changes while developing action-oriented solutions to address parents’ needs in their settings will likely be iterative. Creating an environment where parents feel supported should always be a common effort. One of the evidence- and practice-informed strategies that can be utilized is engagement and partnership with patients and families with lived experience in the creation and delivery of programs [19,20,76].

Professionals who are in positions where they regularly interact with families should be trained and supported in how to be a good resource to families and how they can address some of the families’ needs within the scope of their practice. We collectively need to move past the idea that parents of children with DDs are information providers and ’gatherers’ in clinical interactions or ‘therapists-in-training’ in the rehabilitation process for their child. Our service delivery models need the flexibility to acknowledge parents’ voices and be accommodating of their individual needs (see, for example, King et al.’s (2017) framework of a continuum of family-oriented services [14]) and instruct teams on what type of service might be feasible to implement in their setting for achieving desirable parent outcomes.

## 5. Conclusions

To conclude, as more research and practice examples on parent support programs become available, we will continue to add pieces to the puzzle of parental well-being. The level to which we create ‘villages’ in which parents of children with DDs thrive and how effectively we respond to the much-needed improvement in care provision will depend on the engagement and initiative of all relevant stakeholders and other ‘villagers’, namely researchers, clinicians, healthcare managers, policymakers, community leaders, and most importantly, parents themselves.

## Data Availability

Data sharing is not applicable to this article.

## References

[1] Goldman P.S., Bakermans-Kranenburg M.J., Bradford B., Christopoulos A., Ken P.L.A., Cuthbert C., Duchinsky R., Fox N.A., Grigoras S., Gunnar M.R. (2020). Institutionalisation and deinstitutionalisation of children 2: Policy and practice recommendations for global, national, and local actors. Lancet Child Adolesc. Health.

[2] Krahn G.L., Walker D.K., Correa-De-Araujo R. (2015). Persons with disabilities as an unrecognized health disparity population. Am. J. Public Health.

[3] Rosenbaum P. (2019). Diagnosis in developmental disability: A perennial challenge, and a proposed middle ground. Dev. Med. Child Neurol..

[4] Rosenbaum P. (2020). ‘You have textbooks; we have story books’. Disability as perceived by professionals and parents. Dev. Med. Child Neurol..

[5] Rosenbaum P. (2021). To enhance function, promote children’s development. Dev. Med. Child Neurol..

[6] Rosenbaum P., Novak-Pavlic M., Akhbari Ziegler S., Hadders-Algra M. (2021). Early intervention: What about the family?. Early Detection and Early Intervention in Developmental Motor Disorders.

[7] McCall R.B. (2013). The consequences of early institutionalization: Can institutions be improved?—Should they?. Child Adolesc. Ment. Health.

[8] Areheart B.A. (2008). When disability isn’t just right: The entrenchment of the medical model of disability and the goldilocks dilemma. Indiana Law J..

[9] Brisenden S. (1986). Independent living and the medical model of disability. Disabil. Handicap Soc..

[10] World Health Organization International Classification of Functioning, Disability and Health (ICF). https://www.who.int/standards/classifications/international-classification-of-functioning-disability-and-health.

[11] Kraus de Camargo O., Simon L., Ronen G., Rosenbaum P., Stallinga H., Snyman S., Pederson J. (2019). ICF: A Hands-On Approach for Clinicians and Families.

[12] Ustün T.B., Chatterji S., Bickenbach J., Kostanjsek N., Schneider M. (2003). The International Classification of Functioning, Disability and Health: A new tool for understanding disability and health. Disabil. Rehabil..

[13] Smith J., Cheater F., Bekker H. (2015). Parents’ experiences of living with a child with a long-term condition: A rapid structured review of the literature. Health Expect..

[14] King G., Williams L., Hahn Goldberg S. (2017). Family-oriented services in pediatric rehabilitation: A scoping review and framework to promote parent and family wellness. Child Care Health Dev..

[15] Egilson S.T. (2011). Parent perspectives of therapy services for their children with physical disabilities. Scand. J. Caring Sci..

[16] Piggot J., Hocking C., Paterson J. (2003). Parental adjustment to having a child with cerebral palsy and participation in home therapy programs. Phys. Occup. Ther. Pediatr..

[17] Kayama M., Haight W. (2018). Balancing the stigmatization risks of disability labels against the benefits of special education: Japanese parents’ perceptions. Child. Youth Serv. Rev..

[18] Buljevac M., Majdak M., Leutar Z. (2012). The stigma of disability: Croatian experiences. Disabil. Rehabil..

[19] Miller L., Nickson G., Pozniak K., Khan D., Imms C., Ziviani J., Cross A., Martens R., Cavalieros V., Rosenbaum P. (2022). ENabling VISions and Growing Expectations (ENVISAGE): Parent reviewers’ perspectives of a co-designed program to support parents raising a child with an early-onset neurodevelopmental disability. Res. Dev. Disabil..

[20] Miller L., Imms C., Cross A., Pozniak K., O’Connor B., Martens R., Cavalieros V., Babic R., Novak-Pavlic M., Rodrigues M. (2022). Impact of ‘early intervention’ parent workshops on outcomes for caregivers of children with neurodisabilities: A mixed-method study. Disabil. Rehabil..

[21] Ylvén R., Bjorck-Akesson E., Granlund M. (2006). Literature Review of Positive Functioning in Families with Children with a Disability. J. Policy Pract. Intellect. Disabil..

[22] Kandel I., Merrick J. (2003). The birth of a child with disability. Coping by parents and siblings. Sci. World J..

[23] Lai W.W., Goh T.J., Oei T.P.S., Sung M. (2015). Coping and Well-Being in Parents of Children with Autism Spectrum Disorders (ASD). J. Autism Dev. Disord..

[24] Stuart M., Mcgrew J. (2009). Caregiver Burden after Receiving a Diagnosis of Autism Spectrum Disorder. Res. Autism Spectr. Disord..

[25] Beighton C., Wills J. (2017). Are Parents Identifying Positive Aspects to Parenting Their Child with an Intellectual Disability or Are They Just Coping? A Qualitative Exploration. J. Intellect. Disabil..

[26] Cuzzocrea F., Murdaca A., Costa S., Filippello P., Larcan R. (2015). Parental Stress, Coping Strategies and Social Support in Families of Children with a Disability. Child Care Pract..

[27] Rajan A., Srikrishna G., Romate J. (2018). Resilience and Locus of Control of Parents Having a Child with Intellectual Disability. J. Dev. Phys. Disabil..

[28] Taunt H.M., Hastings R.P. (2002). Positive Impact of Children with Developmental Disabilities on Their Families: A Preliminary Study. Educ. Train. Ment. Retard. Dev. Disabil..

[29] Gupta A., Singhal N. Positive Perceptions in Parents of Children with Disabilities. https://www.semanticscholar.org/paper/POSITIVE-PERCEPTIONS-IN-PARENTS-OF-CHILDREN-WITH-Gupta-Singhal/90f3fe454e718aa5598c8ee6579b37c4ad42befc.

[30] Scorgie K., Wilgosh L., Sobsey D., McDonald J. (2001). Parent Life Management and Transformational Outcomes When a Child Has Down Syndrome. Int. J. Spec. Educ..

[31] Stainton T., Besser H. (2009). The Positive Impact of Children with Intellectual Disability on the Family. J. Intellect. Dev. Disabil..

[32] Skotko B.G., Levine S.P., Goldstein R. (2011). Self-Perceptions from People with Down Syndrome. Am. J. Med. Genet. Part A.

[33] Horsley S., Oliver C. (2015). Positive Impact and Its Relationship to Well-Being in Parents of Children with Intellectual Disability: A Literature Review. Int. J. Dev. Disabil..

[34] Cohn L.N., Pechlivanoglou P., Lee Y., Mahant S., Orkin J., Marson A., Cohen E. (2020). Health Outcomes of Parents of Children with Chronic Illness: A Systematic Review and Meta-Analysis. J. Pediatr..

[35] Brehaut J.C., Garner R.E., Miller A.R., Lach L.M., Klassen A.F., Rosenbaum P.L., Kohen D.E. (2011). Changes over Time in the Health of Caregivers of Children with Health Problems: Growth-Curve Findings from a 10-Year Canadian Population-Based Study. Am. J. Public Health.

[36] Brehaut J.C., Guèvremont A., Arim R.G., Garner R.E., Miller A.R., McGrail K.M., Brownell M., Lach L.M., Rosenbaum P.L., Kohen D.E. (2019). Changes in Caregiver Health in the Years Surrounding the Birth of a Child with Health Problems: Administrative Data from British Columbia. Med. Care.

[37] Brehaut J.C., Kohen D.E., Garner R.E., Miller A.R., Lach L.M., Klassen A.F., Rosenbaum P.L. (2009). Health among Caregivers of Children with Health Problems: Findings from a Canadian Population-Based Study. Am. J. Public Health.

[38] Brehaut J.C., Kohen D.E., Raina P., Walter S.D., Russell D.J., Swinton M., O’Donnell M., Rosenbaum P. (2004). The Health of Primary Caregivers of Children with Cerebral Palsy: How Does It Compare with That of Other Canadian Caregivers?. Pediatrics.

[39] Lach L.M., Kohen D.E., Garner R.E., Brehaut J.C., Miller A.R., Klassen A.F., Rosenbaum P.L. (2009). The Health and Psychosocial Functioning of Caregivers of Children with Neurodevelopmental Disorders. Disabil. Rehabil..

[40] Raina P., O’Donnell M., Rosenbaum P., Brehaut J., Walter S., Russell D., Swinton M., Zhu B., Wood E. (2005). The Health and Well-Being of Caregivers of Children with Cerebral Palsy. Pediatrics.

[41] Cohen E., Horváth-Puhó E., Ray J.G., Pedersen L., Ehrenstein V., Adler N., Vigod S., Milstein A., Sørensen H.T. (2018). Cardiovascular Disease among Women Who Gave Birth to an Infant with a Major Congenital Anomaly. JAMA Netw. Open.

[42] Cohen E., Horváth-Puhó E., Ray J.G., Pedersen L., Adler N., Ording A.G., Wise P.H., Milstein A., Sørensen H.T. (2016). Association Between the Birth of an Infant with Major Congenital Anomalies and Subsequent Risk of Mortality in Their Mothers. JAMA.

[43] Czupryna K., Nowotny-Czupryna O., Nowotny J. (2014). Back pain in mothers of cerebral palsied children. Ortop. Traumatol. Rehabil..

[44] Eda T., Tülin D. (2008). Factors affecting low back pain in mothers who have disabled children. J. Back Musculoskelet. Rehabil..

[45] Azad G., Blacher J., Marcoulides G.A. (2013). Mothers of Children with Developmental Disabilities: Stress in Early and Middle Childhood. Res. Dev. Disabil..

[46] Bonis S. (2016). Stress and Parents of Children with Autism: A Review of Literature. Issues Ment. Health Nurs..

[47] Hastings R.P., Kovshoff H., Brown T., Ward N.J., Espinosa F.D., Remington B. (2005). Coping Strategies in Mothers and Fathers of Preschool and School-Age Children with Autism. Autism Int. J. Res. Pract..

[48] Woodman A.C. (2014). Trajectories of Stress among Parents of Children with Disabilities: A Dyadic Analysis. Fam. Relat..

[49] Beckman P.J. (1991). Comparison of Mothers’ and Fathers’ Perceptions of the Effect of Young Children with and without Disabilities. Am. J. Ment. Retard..

[50] Oelofsen N., Richardson P. (2006). Sense of Coherence and Parenting Stress in Mothers and Fathers of Preschool Children with Developmental Disability. J. Intellect. Dev. Disabil..

[51] Sloper P., Turner S. (1993). Risk and Resistance Factors in the Adaptation of Parents of Children with Severe Physical Disability. J. Child Psychol. Psychiatry.

[52] Findler L., Klein Jacoby A., Gabis L. (2016). Subjective Happiness among Mothers of Children with Disabilities: The Role of Stress, Attachment, Guilt and Social Support. Res. Dev. Disabil..

[53] Heiman T., Berger O. (2008). Parents of Children with Asperger Syndrome or with Learning Disabilities: Family Environment and Social Support. Res. Dev. Disabil..

[54] Dey N., Amponsah B. (2020). Sources of Perceived Social Support on Resilience amongst Parents Raising Children with Special Needs in Ghana. Heliyon.

[55] Kuo D.Z., Cohen E., Agrawal R., Berry J.G., Casey P.H. (2011). A National Profile of Caregiver Challenges among More Medically Complex Children with Special Health Care Needs. Arch. Pediatr. Adolesc. Med..

[56] Centers for Disease Control and Prevention (2022). Data and Statistics for Cerebral Palsy. https://www.cdc.gov/ncbddd/cp/data.html.

[57] Risdal D., Singer G.H.S. (2004). Marital Adjustment in Parents of Children with Disabilities: A Historical Review and Meta-Analysis. Res. Pract. Pers. Sev. Disabil..

[58] Freedman B.H., Kalb L.G., Zablotsky B., Stuart E.A. (2012). Relationship Status among Parents of Children with Autism Spectrum Disorders: A Population-Based Study. J. Autism Dev. Disord..

[59] Namkung E.H., Song J., Greenberg J.S., Mailick M.R., Floyd F.J. (2015). The Relative Risk of Divorce in Parents of Children with Developmental Disabilities: Impacts of Lifelong Parenting. Am. J. Intellect. Dev. Disabil..

[60] Urbano R.C., Hodapp R.M. (2007). Divorce in Families of Children with Down Syndrome: A Population-Based Study. Am. J. Ment. Retard..

[61] Stein A., Pearson R.M., Goodman S.H., Rapa E., Rahman A., McCallum M., Howard L.M., Pariante C.M. (2014). Effects of Perinatal Mental Disorders on the Fetus and Child. Lancet.

[62] Garner R.E., Arim R.G., Kohen D.E., Lach L.M., Mackenzie M.J., Brehaut J.C., Rosenbaum P.L. (2013). Parenting Children with Neurodevelopmental Disorders and/or Behaviour Problems. Child Care Health Dev..

[63] Kuo D.Z., Houtrow A.J., Arango P., Kuhlthau K.A., Simmons J.M., Neff J.M. (2012). Family-Centered Care: Current Applications and Future Directions in Pediatric Health Care. Matern. Child Health J..

[64] Reupert A., Straussner S.L., Weimand B., Maybery D. (2022). It Takes a Village to Raise a Child: Understanding and Expanding the Concept of the “Village”. Front. Public Health.

[65] Baumbusch J., Mayer S., Sloan-Yip I. (2018). Alone in a Crowd? Parents of Children with Rare Diseases’ Experiences of Navigating the Healthcare System. J. Genet. Couns..

[66] Ryan C., Quinlan E. (2018). Whoever Shouts the Loudest: Listening to Parents of Children with Disabilities. J. Appl. Res. Intellect. Disabil..

[67] Baumgardner D.J. (2019). Social Isolation Among Families Caring for Children with Disabilities. J. Patient-Centered Res. Rev..

[68] Currie G., Szabo J. (2020). Social Isolation and Exclusion: The Parents’ Experience of Caring for Children with Rare Neurodevelopmental Disorders. Int. J. Qual. Stud. Health Well-Being.

[69] Greeff A., Nolting C. (2013). Resilience in Families of Children with Developmental Disabilities. Fam. Syst. Health.

[70] Kapp L., Brown O. (2011). Resilience in Families Adapting to Autism Spectrum Disorder. J. Psychol. Africa.

[71] Watson S.L., Hayes S.A., Radford-Paz E. (2011). “Diagnose Me Please!” A Review of Research about the Journey and Initial Impact of Parents Seeking a Diagnosis of Developmental Disability for Their Child. Int. Rev. Res. Dev. Disabil..

[72] National Collaboration Centre for Determinants of Health Vulnerable Populations. https://nccdh.ca/glossary/entry/vulnerable-populations.

[73] Doig J.L., McLennan J.D., Urichuk L. (2009). ‘Jumping through Hoops’: Parents’ Experiences with Seeking Respite Care for Children with Special Needs. Child Care Health Dev..

[74] Landry M.D., Jaglal S., Wodchis W.P., Raman J., Cott C.A. (2008). Analysis of Factors Affecting Demand for Rehabilitation Services in Ontario, Canada: A Health-Policy Perspective. Disabil. Rehabil..

[75] Qi C.Y., Wang Y. (2021). Why Is Rehabilitation Assistance Policy for Children with Disabilities Deviated in Supply-Demand? A Case Study in Mainland China. Front. Public Health.

[76] Pozniak K., Cross A., Babic R., Cavalieros V., Martens R., Rosenbaum P., Imms C., Novak-Pavlic M., Balram A., Hughes D. (2022). Co-development of the ENVISAGE-Families Programme for Parents of Children with Disabilities: Reflections on a Parent-Researcher Partnership. Aust. Occup. Ther. J..

